# Objective assessment of tinnitus laterality

**DOI:** 10.1371/journal.pone.0325903

**Published:** 2025-06-16

**Authors:** Mehrnaz Shoushtarian, Jamal Esmaelpoor, Michelle M.G. Bravo, James B. Fallon

**Affiliations:** 1 The Bionics Institute, Fitzroy, Victoria, Australia; 2 Medical Bionics Department, The University of Melbourne, Melbourne, Australia; 3 Department of Otolaryngology, The University of Melbourne, Melbourne, Australia; The Affiliated Hospital of Zhejiang Chinese Medical University, CHINA

## Abstract

Tinnitus is a condition which involves hearing sounds not present externally. This common condition can lead to a range of symptoms, including depression, resulting in a severe impact on quality of life. There are currently no reliable treatments for tinnitus. One factor hindering development of treatments is the lack of identified subtypes of tinnitus with different underlying patterns of neural activity to enable more personalised treatments and more accurate monitoring of treatment effects. It has been suggested that the perceived laterality of tinnitus, i.e., whether the sound is perceived unilaterally or bilaterally, characterizes tinnitus subtypes with different underlying neural changes. Our previous work showed sensitivity of a non-invasive brain imaging technique called functional near-infrared spectroscopy (fNIRS), to tinnitus-related changes in brain activity. In this study we aimed to investigate differentiating unilateral and bilateral tinnitus using fNIRS recordings and functional network analysis. We performed fNIRS recordings on 18 individuals with unilateral tinnitus (11 left-sided and 7 right-sided), 26 individuals bilateral tinnitus and 18 controls. fNIRS signals were recorded at rest and in response to auditory and visual stimuli. Using network analysis applied to fNIRS recordings, we derived modules characterized by strong connectivity among channels within a module and weak connectivity among channels in different modules. We then calculated two measures, Module Laterality and Modified Module Laterality, to quantify asymmetry in modules. Our findings showed significant difference in Module Laterality in individuals with unilateral tinnitus compared to both bilateral tinnitus and controls. Within the unilateral tinnitus group, Modified Module Laterality showed significant difference between individuals who experienced left-sided tinnitus compared to right-sided tinnitus. Differentiating tinnitus with distinct laterality precepts has the potential to assist in developing and monitoring relevant treatments by revealing neural mechanisms related to each subtype.

## Introduction

Tinnitus – the perception of unwanted sounds which are not present externally – is a debilitating condition which affects 10–20% of adults and can severely impact quality of life, leading to symptoms such as depression and inability to work or sleep [1]. Despite its prevalence, there are no reliable treatments for tinnitus – with some treatments providing symptomatic relief for some people and not others with no clear explanation of why. A critical factor hindering development of treatments is the lack of identified subtypes of tinnitus. An objective assessment of tinnitus that can identify subtypes of tinnitus with different patterns of neural activity would enable more personalised treatments and more accurate monitoring of treatment effects. As an example, in most neuromodulation studies for tinnitus where techniques such as transcranial magnetic stimulation are used, the left auditory cortex or dorsolateral prefrontal cortex (DLPFC) are targeted [2]. However, there are patients who benefit from stimulation to the DLPFC alone, likely due to different underlying brain changes. Identifying subtypes of tinnitus with different patterns of neural activity can therefore assist in more personalised application of treatments such as brain stimulation.

Research suggests that the perceived sound location of tinnitus, i.e., whether the sound is perceived unilaterally or bilaterally, characterizes tinnitus subtypes with different underlying neural changes [3]. In a review of studies on tinnitus laterality, tinnitus was reported to occur bilaterally in 48.8% of cases, on the left side in 28% and right side in 23.2% [4].

Laterality of tinnitus has been associated with asymmetry in hearing loss where different tinnitus perceptions were related to differences in hemispheric responsiveness to changes in neural activity following hearing loss [5]. A study of 62 participants found those with greater hearing loss in the right ear reported right-sided tinnitus whereas individuals with greater hearing loss in the left ear reported their tinnitus as heard bilaterally or mostly on the right.

The underlying neural changes which lead to each of these tinnitus subtypes are not always clearly defined as comparison of hemispheric activity in the auditory cortex has shown differences which have been found in both individuals with tinnitus and controls, suggesting asymmetry in left and right auditory cortex activity is not associated with tinnitus [6].

Studies have also investigated activity in non-auditory regions related to tinnitus laterality. A study which compared electroencephalography (EEG) in individuals with unilateral and bilateral tinnitus suggested a network associated with tinnitus localisation consisting of the auditory cortex, angular gyrus, parahippocampal area and superior premotor cortex [3]. Vanneste et al. performed resting-state EEG recordings (100 2-s epochs) in individuals with unilateral tinnitus and controls [7]. Their study showed a group of individuals with left-sided tinnitus showed higher gamma activity (30.5–44 Hz) in the right parahippocampal area compared to controls [7]. Similarly, comparison of participants with right-sided tinnitus to controls, showed higher gamma activity in the left parahippocampal area. Gamma-band activity in the auditory cortex was not significantly different in left- and right-sided patients with tinnitus [7].

A study investigating power spectral density derived from 5-minute resting state EEG recordings in patients with tinnitus found higher EEG absolute power in the delta and theta bands (typically 0.5–4 Hz and 4–7 Hz respectively) in bilateral tinnitus compared to unilateral tinnitus [8].

Functional near-infrared spectroscopy (fNIRS), a non-invasive brain imaging technique, uses near-infrared light to monitor changes in blood oxygenation levels. Using a montage of light sources and detectors placed on a cap, changes in oxygenated (HbO) and de-oxygenated haemoglobin concentrations (HbR) can be measured. Our previous work demonstrated fNIRS signal features can differentiate tinnitus from controls and tinnitus at different severity levels [9,10]. Using fNIRS and network analysis we also assessed changes in tinnitus severity in a group of cochlear implant users with tinnitus whose perception of tinnitus changed with use of their implant [11]. In this group of participants, we showed changes in subjective ratings of tinnitus loudness and annoyance with the cochlear implant turned on and off, correlated strongly with changes in node strength and diversity coefficient, two network measures derived from fNIRS recordings which quantify neural synchrony. These measures were used as changes to neural synchrony have been reported in tinnitus studies [12]. Node strength quantifies how connected an individual fNIRS channel is with the rest of the network, while diversity coefficient characterizes how a node’s connectivity is distributed within and between network modules [13].

In this study we aimed to investigate the use of functional network analysis on fNIRS recordings, to differentiate unilateral and bilateral tinnitus and additionally, right-sided and left-sided tinnitus in the unilateral group. We compared fNIRS recordings from individuals with unilateral tinnitus (right or left-sided), bilateral tinnitus and a group of controls, using two measures- Module Laterality and Modified Module Laterality, to quantify asymmetry in modules identified in fNIRS cortical networks [14]. Differentiating subtypes of tinnitus with different laterality precepts has the potential to reveal neural mechanisms critical for developing and monitoring relevant treatments.

## Methods

### Participants

This study was approved by the Royal Victorian Eye and Ear Hospital Human Research Ethics Committee (project number 17/1332 H/22). All participants provided written informed consent. Twenty-six participants who experienced bilateral tinnitus and 18 with unilateral tinnitus (11 left-sided and 7 right-sided) were recruited via advertisement through local audiology clinics and Bionics Institute social media. All participants with tinnitus experienced subjective tinnitus with the sound heard only by the person experiencing the condition. No participants with objective tinnitus (sound heard by others, e.g., clinician) were included. Eighteen adults with no history of tinnitus, neurological or hearing disorders were also recruited. The data presented is part of a larger study investigating objective assessment of tinnitus using fNIRS [9,10]. Data used in this study was collected between 10/11/2017 and 28/5/2024. Participants in the three groups (unilateral tinnitus, bilateral tinnitus and controls) were matched for age, gender and hearing thresholds (Table 1). Testing was performed during a single session and in total lasted around 1.5 hours.

**Table 1:** Participant demographics.

	Controls	Unilateral Tinnitus	Bilateral Tinnitus	Group Comparison
No. of participants	18	18	26	
Gender (male: female)	9:9	10:8	12:14	$\chi^{2}=0.38,\;p=0.83$
Age, mean (SD)	65.4 (12.7)	63.5 (11.6)	60.0 (14.4)	$\chi^{2}=2.28,\;p=0.32$
Handedness	R: 18	R:16 L: 1 Both: 1	R: 23 L: 3	$\chi^{2}=3.90,\;p=0.42$
THI, mean (SD)	N/A	50.2 (28.8)	39.5 (24.5)	F(1,42)=1.78,p=0.19
Tinnitus loudness, mean (SD)	N/A	6.0 (3.0)	5.8 (2.5)	F(1,42)=0.05,p=0.83
Tinnitus annoyance, mean (SD)	N/A	5.8 (3.4)	5.0 (3.1)	F(1,42)=0.7,p=0.41
Tinnitus duration (years), mean (SD)	N/A	11.4 (10.8)	13.5 (12.1)	F(1,42)=0.34,p=0.56
Tinnitus laterality	N/A	R: 7 L: 11	N/A	N/A
Hearing thresholds (dB HL), mean (SD)	L ear: 25.6 (10.3) R ear: 25.9 (10.5)	**R-sided tinnitus** L ear: 21.4 (11.1) R ear: 23.2 (12.9) **L-sided tinnitus** L ear: 35.5 (19.6) R ear: 23.3 (7.6)	L ear: 29.0 (17.6) R ear: 24.8 (12.6)	

THI: Tinnitus Handicap Inventory; R: right, L: left; Tinnitus duration: length of time patients have experienced tinnitus.

To obtain a measure of tinnitus severity, participants completed the Tinnitus Handicap Inventory (THI) [15]. The THI includes 25 items which score the severity of tinnitus on a scale of 0–100. Participants were also asked to rate the loudness and annoyance of their tinnitus on a scale of 0–10. Pure tone audiometry was performed on all participants at frequencies of 0.25, 0.5, 1, 2, 4, and 8 kHz. Demographic and clinical data are presented in Table 1. Methods presented are based on our previous studies [9] and described further below.

### fNIRS recordings

**Fig 1:**
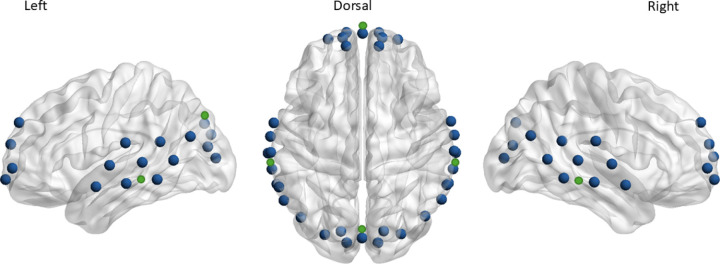
fNIRS recording montage. fNIRS montage covering frontal, left and right temporal and occipital regions. Green circles demonstrate short channels.

fNIRS recordings were performed using a multi-channel continuous wave NIRScout or NIRSport2 device (NIRx Medical Technologies LLC) with 760 and 850 nm wavelengths. Data from nine individuals was collected using NIRSPort2 (controls n = 1, unilateral tinnitus n = 3 and bilateral tinnitus n = 5). The same protocols were used on both devices. Our fNIRS montage consisted of 16 light sources and 16 detectors placed over frontal, temporal and occipital regions with each source-detector pair forming a recording channel (Fig 1). Source-detector pairs placed 30 mm apart formed long channels. Short channels were formed by sources and detectors placed 11 mm apart in frontal, temporal and occipital regions to record scalp and skull systemic signals. Short channels signals were then used to remove systemic artefacts from long channels. The montage was designed using NIRSite software (NIRx Medical Technologies LLC) and an ICBM-152 head model. NIRSite provides MNI coordinates corresponding to channels. These coordinates were used in AtlasViewer software [16] to determine brain regions corresponding to the different channel locations.

fNIRS signals were recorded at rest and in response to auditory and visual stimuli as detailed below.

### Auditory and visual stimuli

Audiometric insert earphones (ER-3A insert earphone, E-A-RTONETM 165 GOLD, USA) were used to deliver 15-second segments of pink noise binaurally, at 65 dB Sound Pressure Level (SPL). Auditory stimuli were calibrated using a Norsonic sound level metre (Norsonic AS, Norway).

To apply visual stimuli, circular checkerboard patterns were displayed with a pattern reversal rate of 7.5 Hz. The auditory and visual stimuli have been used in our previous work on individuals with tinnitus and controls and have shown reliable responses [9,10]. Evoked recordings are commonly used in functional neuroimaging studies of tinnitus [17]. These auditory and visual stimuli showed significantly reduced fNIRS responses in a group of individuals with tinnitus compared to controls [9]. The smaller responses were likely due to increased spontaneous neural activity in auditory regions and the effects on visual regions due to auditory- visual pathways [9]. Both stimuli types were presented using Presentation Software (Neurobehavioral Systems, USA).

### Experimental protocol

Recordings were performed with participants sitting on a comfortable chair in a sound-treated booth. Six-minute resting-state recordings were performed with participants asked to sit without moving, with their eyes closed. Recordings were then performed in response to auditory and visual stimuli. Stimuli included 15-second blocks of sound or a checkerboard pattern presented at random, with no more than two blocks of the same type in a row. Ten trials of each stimulus type were applied with 20 or 25-second non-stimulus intervals in between.

### Data analysis

#### Pre-processing.

**Fig 2:**
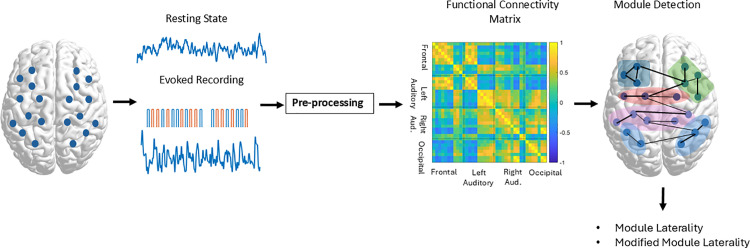
Overview of data analysis. fNIRS data was collected at rest and in response to auditory and visual stimuli (15-second blocks of pink noise or a checkerboard pattern). Arbitrary montage shown for clearer depiction of modules (study montage shown in Fig 1). Ten trials of each stimulus type were collected with 20 or 25-second non-stimulus intervals in between. Three functional connectivity matrices were generated per participant from resting data, concatenated auditory response trials and concatenated visual response trials. Module detection was performed using the Louvain algorithm and laterality of modules was quantified.

An overview of the data analysis is shown in Fig 2. Data processing was performed in Matlab 2023b (Mathworks, USA). The NIRS Brain AnalyzIR Toolbox [18] and custom coded Matlab scripts were used to pre-process fNIRS signals. The Scalp Coupling Index (SCI) was used to identify channels with poor contact with the scalp [19]. The SCI quantifies heart rate activity in the two fNIRS waveforms (760 and 850 nm) filtered in the heart rate range of 0.5–1.8 Hz. Signals from channels with good contact with the skin contain heart rate data within both fNIRS waveforms and show high correlation. All participants in the study had less than 8 channels with SCI < 0.4.

Remaining signals were converted to optical density, corrected for motion artefacts and converted to HbO and HbR concentration using the Modified Beer-Lambert Law [20]. Motion artefact correction was performed using the temporal derivative distribution repair (TDDR) algorithm which corrects sudden shifts in signal amplitude [21]. Extracerebral signals in long channels were removed by regressing systemic signals recorded using short channels [22]. HbO and HbR signals were then band-pass filtered between 0.03–0.4 Hz using zero-phase 8th order Butterworth high-pass and low-pass filters respectively. Finally, a general linear model (GLM) analysis was performed to mitigate the transient effect of evoked responses using canonical hemodynamic response function (HRF)-convolved task regressor. The residual signals after removing the transient effects were considered for creating the functional connectivity networks during different tasks [23,24]. HbO signals were further analysed as previous studies have shown more robust connectivity patterns compared to those obtained from HbR signals [25].

#### Functional connectivity and network analysis.

Connectivity matrices for both resting-state and evoked recordings were derived for each person. To generate matrices for resting-state recordings, Pearson’s correlations between HbO time series from each channel-pair were calculated. For evoked recordings, a time-window around each of the 10 trials from the same stimulus type were concatenated as a time-series, and Pearson’s correlations used as above. The time-window was set from t = 0 to t = 22 seconds relative to stimulus onset. Functional connectivity network measures were then derived from the connectivity matrices, as described below. Asymmetry in the networks was quantified using two measures of laterality described below.

#### Module detection.

Nodes or channels in brain networks form modules which are characterized by strong connectivity among channels within a module, and weak connectivity among channels in different modules [26]. Similar to our previous fNIRS study on cochlear implant users with tinnitus [11], we used the Louvain algorithm to derive modules from functional connectivity matrices. The algorithm initially assigns each channel to a distinct module. Channels are then merged at random into existing modules, to maximise the modularity of the identified modules. The channels are selected for merging at random, therefore the final modules can depend on the order in which channels are chosen and merged. To consider this variability, we ran the algorithm 50 times and averaged the module laterality measure (described below) for detected modules at each iteration.

#### Module laterality.

We used *Module Laterality* to quantify the extent to which channels in an identified module were interhemispheric versus mainly located in one hemisphere. The laterality of a single module within a network is defined as [14]:


$$\mathrm{Module}\;\mathrm{Laterality}=\frac{\left|N_{r}-N_{l}\right|}{N_{t}},\;$$ (1)

where $N_{r}$ and $N_{l}$ are the number of nodes in the right and left hemispheres respectively. $N_{t}$ refers to the total number of nodes in the module. Module Laterality ranges between zero and one. Zero indicates channels in the module are evenly distributed between the two hemispheres, and one indicates the channels are in one hemisphere only. Module Laterality values were averaged to obtain a single Module Laterality value for each person.

Participants in the unilateral tinnitus group perceived their tinnitus either on the right side or left side. To assess laterality in each of these sub-groups, we modified equation (1), by removing the *absolute* function:


$$\mathrm{Modified}\;\mathrm{Module}\;\mathrm{Laterality}=\frac{N_{r}-N_{l}}{N_{t}},\;$$ (2)

This definition provides a way to add polarity to the previous definition of laterality. When a module has more nodes in the right hemisphere compared to the left, the new measure is positive. In contrast, when a module has more nodes in the left hemisphere compared to the right, the new measure is negative.

Modified Module Laterality values were averaged to obtain a single value for each person.

### Statistical analysis

Group differences in Module Laterality and Modified Module Laterality were assessed using one-way analysis of variance (ANOVA) or the Kruskal-Wallis test where assumptions of normality were not met. Post hoc analysis was performed using Tukey tests. If the assumption of homogeneity of variance was not met, Welch’s ANOVA was applied and Games Howell post hoc analysis for unequal variances, was performed. To assess the effect of device type (NIRScout or NIRSPort2) on group differences, two-way ANOVA with group and device as factors was applied. Within the tinnitus groups, the effect of tinnitus severity as assessed by THI, loudness and annoyance ratings as well as duration of tinnitus, device type and asymmetry in hearing thresholds were considered using multiple regression. Asymmetry in hearing thresholds were considered by calculating the difference between left and right ear thresholds and including as a factor. Multi-collinearity between factors was tested using the variation inflation factor and was not detected.

## Results

Summary demographics and clinical data are shown in Table 1. There was no significant group difference in mean age or gender (Table 1). There was no significant difference in hearing thresholds averaged in right ear (F(2,59)=0.39,p=0.68) or hearing thresholds averaged in left ear (F(2,59)=0.25,p=0.78). Comparison of left and right hearing thresholds showed no significant group x side interactions (F(2,118)=0.61,p=0.55).

The unilateral and bilateral tinnitus groups showed no significant difference between THI scores, duration of tinnitus, loudness or annoyance ratings (Table 1).

### Module detection

As described in Methods, to perform module detection, the Louvain algorithm was run 50 times in each of the auditory, visual and rest conditions. The number of modules detected in each condition, across the 50 runs and across participants are reported. In the auditory condition, there were 3–5 modules detected in each group (i.e., same range for controls, right-sided tinnitus, left-sided tinnitus and bilateral tinnitus). In the visual condition, 3–5 modules were detected in the control group, 3–6 in the left-sided tinnitus group, 2–5 in the right-sided tinnitus group and 2–6 in the bilateral tinnitus group. In the rest condition, 2–5 modules were detected in controls, 3–5 in each of the unilateral tinnitus groups and 2–5 modules in the bilateral tinnitus group.

**Fig 3:**
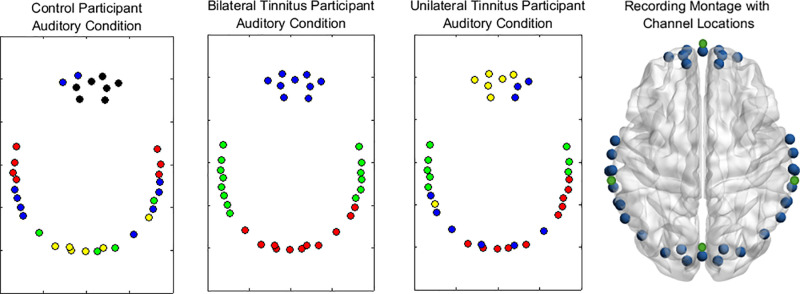
Functional modules. Modules derived using Louvain’s algorithm, shown for representative participants in the control, bilateral tinnitus and unilateral tinnitus groups. Circles indicate fNIRS long channels arranged based on montage shown on right side. Colours show different modules detected.

Fig 3 shows modules detected for a representative participant in each group. Channels shown in the same colour represent a module.

### Module laterality

**Fig 4:**
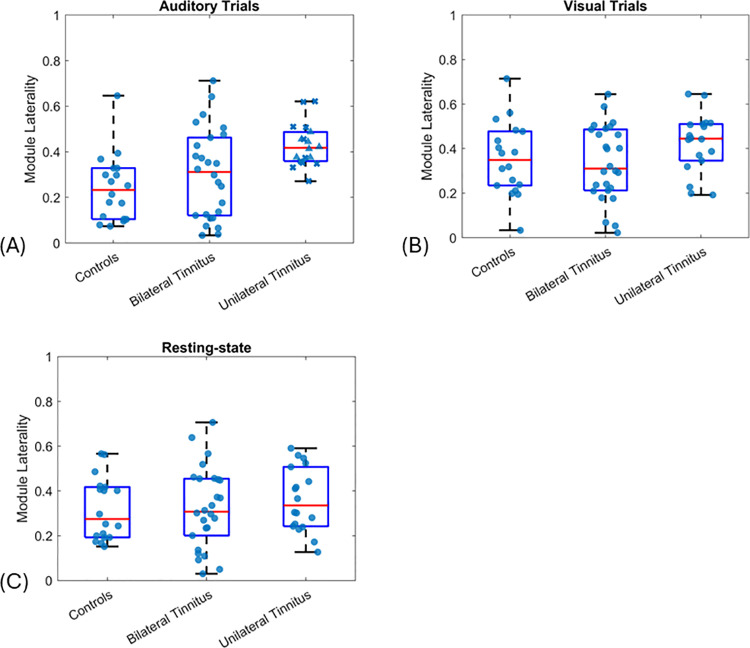
Module Laterality. Values calculated for (A) auditory trials, (B) visual trials and (C) resting state. Blue markers show data points for each participant. For auditory trials (A), triangle markers show right-sided tinnitus and x markers show left-sided tinnitus. Boxes show median, interquartile range and largest/ smallest values.

Module Laterality values for control, bilateral and unilateral tinnitus groups are shown in Fig 4. Values are shown for resting-state recordings and trials in response to auditory or visual stimuli. For auditory trials (Fig 4A), Welch’s one-way ANOVA showed a significant overall group effect (F(1,37.76)=8.16,p=0.02) with higher Module Laterality in the unilateral tinnitus group compared to both controls (p=0.002) and the bilateral tinnitus group (p=0.02). Overall group effect was not significant for visual trials or resting-state recordings (Figs 4B and 4C).

No group differences between controls and bilateral tinnitus were found (Fig 4).

The effect of device (NIRScout or NIRSPort2) on laterality values in the auditory condition, was assessed using two-way ANOVA with factors group and device. Device did not show a significant effect, while the overall group effect remained significant (F(2,58)=6.77,p=0.002), with unilateral tinnitus showing significantly higher Module Laterality compared to controls (p=0.002) and the bilateral tinnitus group (p=0.03).

Notably, Module Laterality was found to be significantly higher in the unilateral tinnitus group.

Multiple regression was used to evaluate the effect of various factors on Module Laterality within the two tinnitus groups. Factors included THI, loudness and annoyance (as measures of tinnitus severity), duration of tinnitus, device type (NIRScout or NIRSPort2) and hearing asymmetry as measured by the difference in left and right hearing thresholds. The effect of group was significant (β=0.12, SE=0.05,p=0.02), and all factors did not show a significant effect, suggesting the difference in Module Laterality was not due to differences in tinnitus characteristics, differences in left and right hearing thresholds or the fNIRS device used.

### Modified module laterality

**Fig 5:**
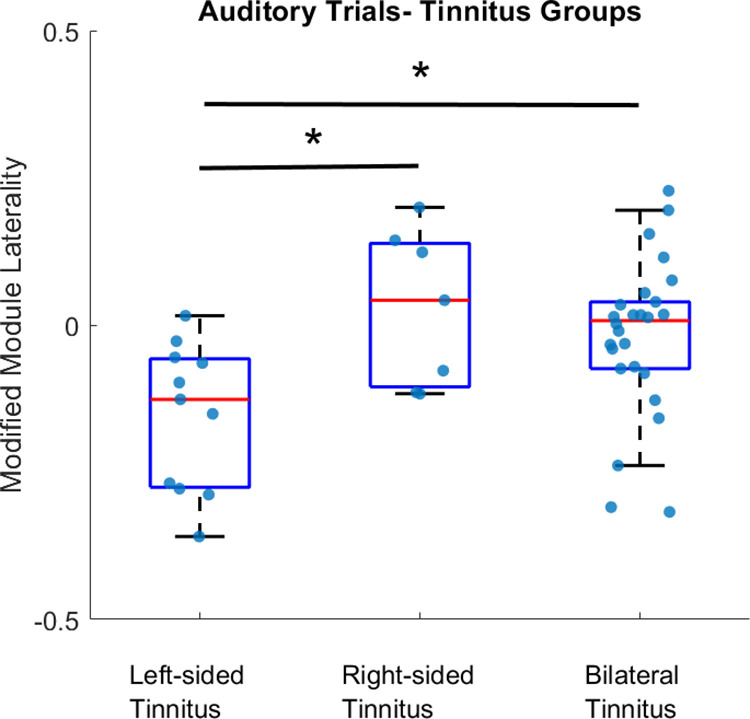
Modified Module Laterality. Values derived from auditory trials for left-sided, right-sided and bilateral tinnitus groups (* indicates p<0.05). Boxes show median, interquartile range and largest/ smallest values. Blue markers show data points for each participant.

To assess differences in individuals in the unilateral tinnitus group based on perceived tinnitus on the right or left side, we used a modified form of Module Laterality (Equation 2). Modified Module Laterality values derived from auditory trials (based on findings above) for left-sided, right-sided and bilateral tinnitus, are shown in Fig 5. Using this measure, differences in the left- and right-sided tinnitus groups can be seen for auditory trials, with a negative median value for the left-sided tinnitus group and positive median value for the right-sided tinnitus group (Fig 5).

A Kruskal-Wallis test showed a significant overall group effect ($\chi^{2}(2)=8.13,p=0.02$) with post-hoc analysis showing a significant difference between left- and right-sided tinnitus (p=0.04) as well as left-sided and bilateral tinnitus (p=0.03).

To assess the effect of THI, duration of tinnitus, loudness, annoyance, device type and asymmetry in hearing thresholds on Modified Module Laterality in the two groups, we used multiple regression. The effect of group remained significant (β=0.1, SE=0.04,p=0.01) with none of the added factors showing a significant effect.

## Discussion

In this study we applied network analysis to fNIRS signals recorded from individuals with unilateral or bilateral tinnitus, and controls. We derived channels forming modules and using a measure called Module Laterality, assessed whether modules contained channels mostly in one hemisphere or spread across both hemispheres. There was a significant difference in Module Laterality in individuals with unilateral tinnitus compared to both bilateral tinnitus and controls. By modifying Module Laterality to quantify whether channels in a module were mostly on the left or right hemisphere, we found a significant difference between individuals who experienced left-sided tinnitus compared to right-sided tinnitus and those with tinnitus perceived bilaterally.

The significant group differences observed were in recordings in response to auditory stimulation. As described below, studies suggest asymmetry in resting state and evoked activity in auditory regions is seen in both individuals with tinnitus and controls and therefore cannot be attributed to tinnitus-related changes in brain activity (e.g., [6]). However tinnitus is known to result from changes in activity in multiple brain networks [27] and therefore our approach in using network analysis is likely more suited to detecting cortical network changes that lead to lateralisation of the perception of tinnitus.

Studies using positron emission tomography (PET) proposed higher resting metabolic activity in the left primary auditory cortex compared to the right in patients with tinnitus, e.g., [28,29]. However, more recently it was shown that asymmetry in auditory regions was also present in individuals without tinnitus [6]. In both controls and individuals with tinnitus, primary auditory cortex activity was found to be higher on the left side, whereas in secondary and associated auditory areas, right side activity was higher [6]. Several studies compared sound-evoked activity in individuals with tinnitus and controls using functional magnetic resonance imaging (fMRI). Symmetrical responses were found in those with bilateral tinnitus whereas those experiencing unilateral tinnitus showed a higher response in the primary auditory cortex ipsilateral to the side tinnitus was perceived [30,31]. In both studies the tinnitus and control groups were not matched for hearing levels which may have confounded findings [17]. In controls, group averaged Modified Module Laterality values resulted in a value of −0.06, showing slight asymmetry which agrees with known asymmetry in brain activity in controls. By assessing sound-evoked network connectivity, we have shown group differences associated with lateralisation of tinnitus.

Group differences in Module Laterality and Modified Module Laterality derived from visual and rest conditions were not significant (Figs 4 and 5), suggesting suitability of the auditory paradigm for assessing laterality in tinnitus.

The laterality measures used in this study quantify how modules are spread across hemispheres which could be affected by known tinnitus-related changes in connectivity. Changes in functional connectivity across hemispheres have been observed in tinnitus [32]. Using fMRI, researchers compared connectivity by calculating Pearson’s correlation coefficient between voxels in one hemisphere and their symmetrical counterparts in the opposite hemisphere. This analysis revealed increased connectivity in tinnitus patients compared to controls, in the middle temporal gyrus, middle frontal gyrus, and superior occipital gyrus. Findings from the study could not be associated with one type of tinnitus in terms of laterality as the study included participants with both unilateral tinnitus and bilateral (12 with predominantly left-sided tinnitus, nine right-sided and seven bilateral or originating in the head).

Shin et al. used resting fMRI, to compare laterality in auditory and non-auditory networks between individuals with unilateral tinnitus (19 right-sided and 19 left-sided) and controls [33]. Laterality of networks were calculated based on the left hemisphere’s functional network map and its corresponding voxels from the right hemisphere. The tinnitus group showed reduced left lateralisation in the auditory network and reduced right lateralisation in the visual network in tinnitus compared to controls. No significant difference was found between left-sided and right-sided tinnitus. In our study, modules were not constrained to be anatomically based and therefore Module Laterality provided an overall quantification of laterality which was increased in unilateral tinnitus and in its modified form was able to differentiate between left-sided and right-sided tinnitus.

The observed increase in Module Laterality in unilateral tinnitus may be due to increased intra-hemispheric synchrony. Our previous work investigated synchrony in tinnitus by assessing distribution of connectivity within and between modules using a measure called diversity coefficient [11]. The study included cochlear implant users who experience tinnitus and whose perception of tinnitus changes with use of their implant. In individuals who reported reduced tinnitus loudness with use of their cochlear implant, higher diversity coefficient or a more even distribution within and between modules was found.

Reduced inter-hemispheric connectivity may also lead to the increase in Module Laterality in unilateral tinnitus. A study on 58 individuals with tinnitus assessed the correlation between laterality of tinnitus and functional asymmetries (e.g., handedness or dichotic listening) [4]. A higher correlation was found between tinnitus laterality and dichotic listening compared to other functional asymmetries. Dichotic listening also showed deficits in individuals with epilepsy who had surgery to separate the left and right hemispheres (severing of the corpus callosum) [34], which would reduce interhemispheric connectivity.

Altered interhemispheric connectivity in tinnitus may also reflect changes in underlying structural connectivity. However, such changes are unclear as some studies investigating tinnitus-related structural changes have reported differences between tinnitus and controls (e.g., [35]) whereas others reported no differences when participants were closely matched for age, gender and hearing threshold [36]. It was suggested that differences in grey matter volume found between tinnitus and controls are likely associated with hearing loss, particularly at thresholds beyond 8 kHz [36]. In our study, groups were matched for hearing loss on both the left and right sides at frequencies up to 8 kHz, making it unlikely that differences in laterality measures are related to hearing loss. However, differences at higher frequencies could potentially contribute to these variations. Future studies could include higher frequencies when assessing hearing loss.

A limitation of our study is the broad definition of laterality we have applied. Participants described their tinnitus as right-sided, left-sided or heard on both sides. Recent work which reviewed reporting of tinnitus localisation recommended more detailed reporting of tinnitus localisation through six categories covering the coronal (left, centre or right) and sagittal (front, ear or back) planes [37]. With larger datasets, more refined localisation of underlying changes in brain activity in tinnitus can be investigated, assisting in improved monitoring of potential treatments. In addition, future studies can investigate brain activity related to laterality of tinnitus in patients who perceive sounds with similar pitch or intensity.

Using two network-based measures of laterality we have shown significant differences in fNIRS recordings in unilateral tinnitus compared to bilateral tinnitus and controls and left- and right-sided tinnitus. Identifying these subtypes of tinnitus based on laterality precepts has the potential to reveal neural mechanisms critical for developing and monitoring relevant treatments. Treatments such as different forms of brain stimulation which can be applied to different brain regions, could be guided by knowledge of the underlying neural changes which need to be targeted.
